# Polyamines control of cation transport across plant membranes: implications for ion homeostasis and abiotic stress signaling

**DOI:** 10.3389/fpls.2014.00154

**Published:** 2014-04-23

**Authors:** Igor Pottosin, Sergey Shabala

**Affiliations:** ^1^Biomedical Centre, Centro Universitario de Investigaciones Biomédicas, University of ColimaColima, Mexico; ^2^School of Land and Food, University of TasmaniaHobart, TAS, Australia

**Keywords:** cytosolic calcium, ion channels, ion pumps, plasma membrane, polyamines, reactive oxygen species, stress, vacuole

## Abstract

Polyamines are unique polycationic metabolites, controlling a variety of vital functions in plants, including growth and stress responses. Over the last two decades a bulk of data was accumulated providing explicit evidence that polyamines play an essential role in regulating plant membrane transport. The most straightforward example is a blockage of the two major vacuolar cation channels, namely slow (SV) and fast (FV) activating ones, by the micromolar concentrations of polyamines. This effect is direct and fully reversible, with a potency descending in a sequence Spm^4+^ > Spd^3+^ > Put^2+^. On the contrary, effects of polyamines on the plasma membrane (PM) cation and K^+^-selective channels are hardly dependent on polyamine species, display a relatively low affinity, and are likely to be indirect. Polyamines also affect vacuolar and PM H^+^ pumps and Ca^2+^ pump of the PM. On the other hand, catabolization of polyamines generates H_2_O_2_ and other reactive oxygen species (ROS), including hydroxyl radicals. Export of polyamines to the apoplast and their oxidation there by available amine oxidases results in the induction of a novel ion conductance and confers Ca^2+^ influx across the PM. This mechanism, initially established for plant responses to pathogen attack (including a hypersensitive response), has been recently shown to mediate plant responses to a variety of abiotic stresses. In this review we summarize the effects of polyamines and their catabolites on cation transport in plants and discuss the implications of these effects for ion homeostasis, signaling, and plant adaptive responses to environment.

## Introduction

Polyamines (PAs) are acknowledged regulators of plant growth, development, and stress responses. In the model plant *Arabidopsis*, changes in the expression of different enzymes of the PAs biosynthesis and respective levels of individual PAs are stress-specific, and these changes mediate stress tolerance (Alcázar et al., 2010); same may be true also for other plants (see below). Polyamines at physiological pH are polycations, bearing from 2 (putrescine, Put) to 4 (spermine, Spm or thersmospermine, tSpm) positive charges. Thus, PAs can stabilize membranes or nucleic acids, binding to their negative surfaces (Galston and Sawhney, 1990; Kusano et al., 2008). They can also act as a source of reactive oxygen species (ROS) but also as ROS scavengers and activators of key of antioxidant enzymes (Kusano et al., 2008; Moschou et al., 2008; Pottosin et al., 2014a). In animal cells PAs affect a variety of plasma membrane (PM) cation channels, acting primarily as pore blockers but in some cases also affecting the channel gating and/or regulation by extra- and intracellular ligands (Drouin and Hermann, 1994; Lopatin et al., 1994; Williams, 1997; Lu and Ding, 1999; Huang and Moczydlowski, 2001; Xie et al., 2005; Ahern et al., 2006). PAs effects on vacuolar channels in plants were revealed and partly reviewed (Pottosin and Muñiz, 2002), but the accumulated experimental evidence for the PAs effects on the plant PM ion channels and pumps was not properly discussed until now. This review is aimed to fill this gap and provide a comprehensive overview on our current knowledge of PA control over cation transport across plant membranes, and its implications for ion homeostasis, signaling, and plant adaptive responses to environment.

## Stress-induced changes in polyamine content and their role in plant adaptive responses to environment

Polyamine levels are strongly modulated by literally every known abiotic factor (see Table 1, for selected examples), often reaching the millimolar level (such as for putrescine; Galston and Sawhney, 1990; Alcázar et al., 2006) under stress conditions. The current consensus is that, rather than being merely collateral effect of stress-induced metabolic changes, these changes are beneficial to plant performance upon stress (Alcázar et al., 2010; Gupta et al., 2013) and therefore represent an important component of plant adaptive mechanisms. Three major lines of evidence support this claim, which can be illustrated for salinity and drought stresses—two key abiotic stresses affecting agricultural crop production around the world. First, externally applied PAs ameliorate stress symptoms. At salt stress, the beneficial effects are due to improved ionic (K^+^/Na^+^) relations (Zhu et al., 2006; Ndayiragije and Lutts, 2007; Roychoudhury et al., 2011; Sharma et al., 2013b) and increased antioxidant activity, both enzymatic (Ozturk and Demir, 2003; Tang and Newton, 2005; Chai et al., 2010) and non-enzymatic, e.g., via proline accumulation (Su and Bai, 2008; Sharma et al., 2013b). Similarly to salinity, PAs improved drought tolerance due to the activation of antioxidant systems, both enzymatic (Kubiś, 2008) and non-enzymatic (such as proline, anthocyanins, and soluble phenolics; Farooq et al., 2009). This reduces the amount of ROS produced (Farooq et al., 2010) and stabilizes membrane structures (Kubiś, 2006). Under natural conditions, PA may also play a beneficial role in mycorrhizal development, contributing to improved plant water status and water use efficiency (Wu et al., 2010). Second, in many cases accumulation of PAs positively correlates with salt (Chattopadhayay et al., 2002; Liu et al., 2006; Mutlu and Bozcuk, 2007) and drought (van der Mescht et al., 1998; Liu et al., 2005), resistance. Third, and maybe the strongest line of evidence came from the experiments with the gain- or loss-of-the-function mutants. Whereas overexpression of enzymes of the PA biosynthesis like arginine decarboxylase, ADC (Roy and Wu, 2001), S-adenosylmethionine synthetase, SAMDC (Waie and Rajam, 2003; Qi et al., 2010), and spermidine synthase, SPDS (Neily et al., 2011) resulted in improved salt tolerance, the loss-of-function mutant of PA biosynthesis genes resulted in reduced stress tolerance in several species (Fariduddin et al., 2013). In Arabidopsis, loss-of-the-function mutants in the synthesis of Spm and thermospermine accumulated more Na^+^ and performed worse than wild type in survival experiments (Alet et al., 2012). Similarly, the introduction of SAMDC gene led to increased polyamine biosynthesis and increased drought tolerance in tobacco (Waie and Rajam, 2003) and rice (Li and Chen, 2000; Peremarti et al., 2009). Over-expression of ADC in Arabidopsis resulted in a transgenic line with enhanced resistance to high osmoticum, dehydration, and long-term drought (Wang et al., 2011). Transgenic Arabidopsis plants displayed a reduced transpiration rate and stomata conductance, hence, a lesser water loss (Alcázar et al., 2010). Conversely, Arabidopsis mutant plants not capable of producing Spm were hypersensitive to drought (Yamaguchi et al., 2007).

**Table 1:** **Stress-induced changes in the level of free polyamines in plants**.

**Species**	**Stress**	**Put**	**Spd**	**Spm**	**References**
Pine	Drought	Up	Up	Up	De Diego et al., 2013
Pepper	Drought	Up^a^	Up^a^	Up^a^	Sziderics et al., 2010
Rice	Drought	Down	Up	Up	Yang et al., 2007
Wheat	Osmotic	Up	Up	Up	Grzesiak et al., 2013
Potato	Osmotic	Down	Down	Down	Li et al., 2005
Bromus	Osmotic	Down	Down	Down	Gicquiaud et al., 2002
Cucumber	Flooding	Up	Up	Up	Shi et al., 2008
Citrus	Flooding	Up	Up^b^	Up^b^	Arbona et al., 2008
Tobacco	Flooding	Up	Up	Steady	Hurng et al., 1994
Rice	Anoxia	Up	Steady or up	Steady or up	Reggiani et al., 1989
Arabidopsis	Heat	Up	Up	Up	Sagor et al., 2013
Tobacco	Heat	Up	Up	Up	Cvikrova et al., 2012
Wheat	Heat	Down	Up	Up	Goyal and Asthir, 2010
Wheat	Cold	Up	Up	Up	Kovacs et al., 2010
Maize	Cold	Up	Steady	Down	Nemeth et al., 2002
Wheat	CO_2_	Down	Up	Up	Högy et al., 2010
Common sage	UV	Up	Up	Up	Radyukina et al., 2010
Scots pine	K^+^ deficit	Up	Steady	Steady	Sarjala, 1996
Arabidopsis	K^+^ deficit	Up	Steady	Steady	Watson and Malmberg, 1996

a In leaves but not root tissues;

b *in sensitive genotype only*.

## Controversies and inconsistencies

While a large body of evidence exists suggesting that changes in PA level and plant adaptive responses to environment are positively correlated, negative, or no correlation were reported as well. No correlation between PA content and drought tolerance was found among contrasting rice cultivars (Do et al., 2013) or even higher PAs levels were reported for drought-sensitive chickpea and beans varieties compared with their tolerant counterparts (Juhasz et al., 1997; Nayyar et al., 2005). Although PAs treated rice plants showed improved K^+^/Na^+^ ratio in shoots, PAs did not protect them against salt. Exogenously applied putrescine (Put) induced a decrease in the shoot water content in the presence of NaCl, while spermidine (Spd) and spermine (Spm) were detrimental for cell membrane stability (Ndayiragije and Lutts, 2006). Low-PA lines of maize appeared to be tolerant to salt stress while high-PA lines were more sensitive (Zacchini et al., 1997). Negative correlation between changes in Put content and salinity stress tolerance was reported in experiments comparing different plants (Zapata et al., 2007). Within six selected species, the most tolerant ones accumulate less Put compared with salt-sensitive ones (Zapata et al., 2008). Thus, it appear the concept “the more PA the better for stress tolerance” does not always held.

To support the above statement, we have tabulated some selected evidence for NaCl-induced changes in the level of free PA in root and leaf tissues of various species (Table 2). Of 23 papers cited, eight reported an increase in the level of all three PA; seven have reported a significant reduction in all PA levels; and eight showed mixed results (e.g., an increase in the level of one specific PA accompanied by the concurrent decrease in the level of another PA). Obviously, aspects such as time- and concentration-dependence of PA synthesis and metabolism, its tissue- and organelle-specificity, and inter-conversion between various types and forms of PA, all should be not ignored.

**Table 2:** **Selected examples of the changes in the level of free polyamines induced by salt stress in plant root and leaf tissues**.

**Species**	**Put**	**Spd**	**Spm**	**References**
Cucumber	Up	Up	Up	Fan et al., 2013
Bean	Down	Down	Down	Shevyakova et al., 2013
Pea	Up	Up	Up	Piterkova et al., 2012
Plantago	Down	Down	Down	Radyukina et al., 2009
Mesembryanthemum	Up	Up	Up	Shevyakova et al., 2006
Mesembryanthemum	Down	Down	Up	Stetsenko et al., 2009
Chickpea	Down	Down	Down	Nayyar et al., 2005
Maize	Up	Up	Up	Rodríguez et al., 2009
Apple	Down	Steady	Down	Liu et al., 2008
Grape	Up	Up	Up	Upreti and Murti, 2010
Bromus	Down	Down	Down	Gicquiaud et al., 2002
Tomato	Up	Up	Up	Botella et al., 2000
Tomato	Down	Down	Down	Aziz et al., 1999
Barley	Up	Up	Up	Zhao et al., 2003
Jojoba	Down	Down	Up	Roussos and Pontikis, 2007
Ginseng	Down	Up	Up	Parvin et al., 2012
Wheat	Down	Up	Up	Reggiani et al., 1994
Lupin	Up	Up	Steady	Legocka and Kluk, 2005
Sunflower	Down	Down	Up	Mutlu and Bozcuk, 2007
Soybean	Down	Down	Steady	Xing et al., 2007
Quinoa	Down	Steady	Up	Ruiz-Carrasco et al., 2011
Sunflower	Down	Down	Up	Mutlu and Bozcuk, 2005
Lettuce	Down	Up	Up	Zapata et al., 2003

Plant adaptive responses to environment are closely and ultimately related to their ability to control intracellular ion homeostasis and regulate ion transport across cellular membrane (Shabala, 2012). Different tissues show different patterns of ion accumulation, with dicots and monocots sometimes displaying contrasting patterns for a distribution of a certain ion (e.g., Na^+^) between different tissues. Understanding of the relative ion accumulation and tissue-specific expression of ion channels and transporters has just started to emerge (Karley et al., 2000; Volkov et al., 2003; Conn and Gilliham, 2010; Gilliham et al., 2011). Plant membranes host hundreds of transport proteins that comprises of ~5% of the entire Arabidopsis genome (Mäser et al., 2001). Some of them are known to be strongly affected by PAs, and PAs can also exert contrasting effects on the same individual ion transporter via diverse mechanisms of action (see below). Thus, the causal role of PA in plant adaptive responses to environment may be established only in the strict context of the tissue- and organelle-specificity.

## Polyamines effects on the vacuolar cation transport

Slow (SV) and fast (FV) vacuolar channels are non-selective cation channels that are ubiquitously and abundantly expressed in higher plant vacuoles (Hedrich et al., 1988; Pottosin and Muñiz, 2002; Hedrich and Marten, 2011; Pottosin and Dobrovinskaya, 2014). SV channels are encoded by the two-pore cation (TPC1) gene (Peiter et al., 2005), whereas the molecular identity of FV channels is still elusive. Both channels conduct a variety of small monovalent cations with a little preference, but SV channels also conduct alkali earth cations like Ca^2+^ and Mg^2+^ (Amodeo et al., 1994; Brüggemann et al., 1999a; Pottosin et al., 2001; Pottosin and Dobrovinskaya, 2014). SV and FV channels only weakly differentiate between K^+^ and Na^+^; this also holds for the case of halophyte plants (Bonales-Alatorre et al., 2013). SV channels are activated by the increase in the cytosolic Ca^2+^, and, with a lower affinity, by Mg^2+^ (Hedrich and Neher, 1987; Ward and Schroeder, 1994; Pottosin et al., 1997; Carpaneto et al., 2001). At the same time, FV channels are inhibited by the micromolar cytosolic Ca^2+^ and Mg^2+^ (Tikhonova et al., 1997; Brüggemann et al., 1999b; Pei et al., 1999). Therefore, one may propose that the contribution of FV and SV currents into the overall tonoplast cation conductance, among other factors, maybe regulated by the cytosolic Ca^2+^. In this model, FV channels are more active at the resting Ca^2+^ levels, whereas SV channels require a very substantial cytosolic Ca^2+^ increase for their activation.

**Figure 1:**
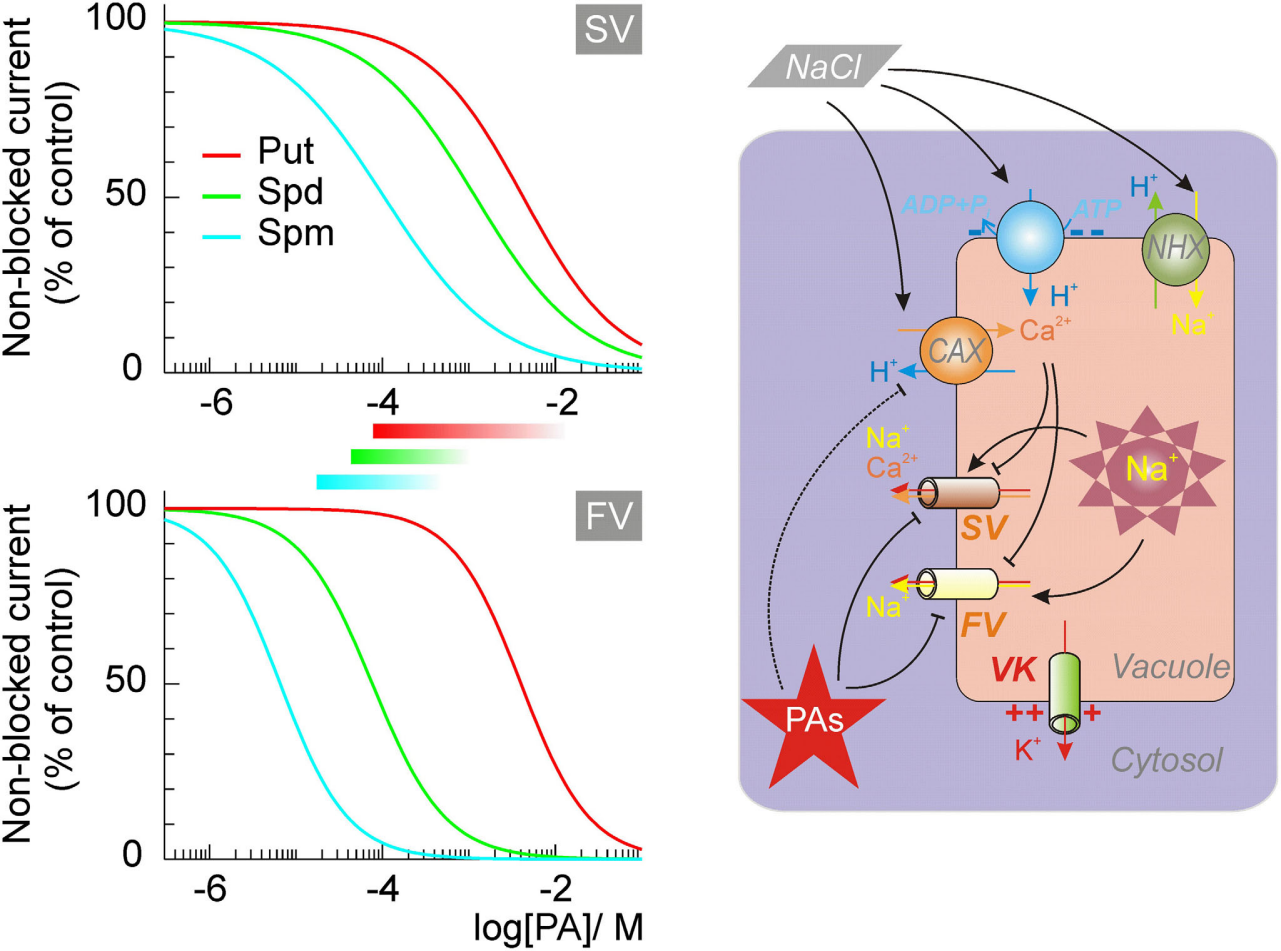
**The dose-dependence of the vacuolar cation channels' block by intracellular polyamines and its implications for the salt stress resistance**. Dose dependence for SV and FV channels at physiologically attainable (zero) tonoplast potential are drawn, using the values of blocking parameters from Brüggemann et al. (1998) and Dobrovinskaya et al., (1999a,b). Approximate ranges for intracellular PAs in plant cells are indicated by bars. At high salinity, efficient vacuolar Na^+^ sequestration is critical for the salt tolerance. This requires the increased Na^+^/H^+^ antiport activity and a decrease of Na^+^ leaks through non-selective FV and SV cation channels. The block by PAs would abolish the FV-mediated current, and strongly suppress the SV current. Continuous operation of the VK, weakly sensitive to PAs, acts as a shunt conductance for the electrogenic H^+^-pump, which fuels the active Na^+^ uptake, and contributes to the recuperation of the salt-induced cytosolic K^+^ loss. Salt stress stimulates expression of the cation-H^+^ antiporters, which may reduce the FV and SV activity via the increase of the luminal Ca^2+^. Over-expression of CAXs is also caused by the inhibition of the Spm^4+^ synthesis. Thus, PAs and vacuolar Ca^2+^ may act as alternative regulators of vacuolar cation channels.

In animal cells, several K^+^ and cation channels, sensitive to Mg^2+^, are also sensitive to PAs (Williams, 1997). Similarly, Mg^2+^-sensitive FV channels were efficiently blocked by micromolar concentrations of Spm and Spd, and by millimolar concentrations of Put (Brüggemann et al., 1998; Dobrovinskaya et al., 1999a). The blockage occurred instantaneously, was dose- but not voltage-dependent, and fully reversible (see Figure 1 and Table 3, for a quantitative description).

**Table 3:** **Summary of polyamine effects on plant ion channels and pumps**.

**Channel or pump**	**Mechanism of the PA action**	**References**
**VACUOLAR CHANNELS**		
SV (TPC1): slow vacuolar (two-pore cation) Ca^2+^-permeable channel	Direct, reversible. Voltage-dependent block from either membrane side	Dobrovinskaya et al., 1999a,b
	Spm (50 μM) > Spd (500 μM) > Put (3 mM)^*^	
FV: fast vacuolar monovalent cation channel	Direct, reversible. Voltage-independent block from the cytosolic side	Brüggemann et al., 1998; Dobrovinskaya et al., 1999a
	Spm (6 μM) > Spd (80 μM) >> Put (4 mM)^*^	
VK (TPK1): vacuolar K^+^ (two-pore K^+^)	Direct (?) Voltage-independent, cytosolic side	Hamamoto et al., 2008
	Spm ~ Spd (~1 mM) > >Put ^**^	
**PLASMA MEMBRANE CHANNELS**		
KIRC: inward rectifying K^+^ channel -Guard cells (KAT1)	Indirect, cytosolic side, V-independent	Liu et al., 2000
	Spm ~ Spd ~ Put (0.5–1 mM)^**^	
-Roots (AKT1)	Indirect, extracellular side, V-independent	Zhao et al., 2007; Zepeda Jazo, 2010
	Spm ~ Spd (~1.5 mM) > Put^**^	
KORC (GORK): outward rectifying K^+^ channel	Indirect, extracellular side, V-independent	Zepeda Jazo, 2010
	Spm ~ Put (~1 mM)^**^	
VI-NSCC: voltage-independent cation channel	Extracellular side, V-independent	
-roots	Spm ~ Spd (~0.4 mM) > Put^**^	Zhao et al., 2007
-leaves	Extracellular side (indirect?)	Shabala et al., 2007a,b
	Spm ~ Put (~0.4 mM)^**^	
ROSIC: weakly voltage-dependent, OH•-induced non-selective conductance	Extracellular PAs act as cofactors for ROSIC activation by OH•	Zepeda-Jazo et al., 2011; Pottosin et al., 2012; Velarde-Buendía et al., 2012
	Spm ~ Spd ~ Put (1 mM)	
**PLASMA MEMBRANE P-TYPE ATPASES**		
ACA: autoinhibited Ca^2+^-ATPAse	Rapid activation of Ca^2+^-pumping	Bose et al., 2011; Zepeda-Jazo et al., 2011; Pottosin et al., 2012; Velarde-Buendía et al., 2012
	Spm ~ Put (0.1–1 mM)	
	Long-term potentiation	Sudha and Ravishankar, 2003
AHA: autoinhibited H^+^-ATPAse	Rapid activation (coupled to Ca^2+^ pump)	Velarde-Buendía, 2013
	Put (1 mM)	
	Rapid activation and/or inhibition (0.1 or mM Spm)	
	Inhibition of the H^+^ pumping; Spm > Spd ~ Put (~1 mM)	Pandolfi et al., 2010
	Activation Spm ~ Spd ~ Put (~1 mM)^**^	Reggiani et al., 1992; Garufi et al., 2007
	Activation via 14-3-3 proteins binding (Spm only, ~0.1 mM)^**^	
	Long-term suppression, lower expression	Janicka-Russak etal., 2010

* *Numbers in brackets are apparent K_d_ from the data, presented in Figure 1*.

** *Numbers in brackets are approximated concentrations of PAs, producing the half-effect*.

*When not marked, simply tested experimental concentrations are given*.

SV channels possess a wide pore, with about 0.7 nm diameter in the narrowest constriction (Pottosin and Schönknecht, 2007). Therefore, it is not surprising that PAs act as permeable pore blockers (Dobrovinskaya et al., 1999a,b). This blockage of SV channels is qualitatively very similar to PA blockage of sodium- (Huang and Moczydlowski, 2001), cyclic nucleotide-gated- (Guo and Lu, 2000), and ryanodine- or acetylcholine-receptor channels (Uehara et al., 1996; Haghighi and Cooper, 1998) in animal cells. The block of SV channels was voltage-dependent, with the affinity increasing at increased positive potential at the side of the PAs application. At higher potentials, however, a relief of the block due to a “punchthrough” phenomenon was observed, when PAs were forced to cross the entire channel pore by a strong electric field. Comparison of the voltage-dependence of the block from cytosolic and vacuolar sides displayed several interesting features. First, it appears that PAs reached a common docking point from either membrane side. Second, electric and physical distance from the pore entrance to this stop point was approximately three times longer from the cytosolic side as compared to that from the vacuolar side. Consequently, the cytosolic part of the pore can adopt single Spm or two Put molecules at a time, whereas the vacuolar part can contain a single blocking molecule at a time, regardless of whether it is Spm, Put or a quaternary ammonium ion (Dobrovinskaya et al., 1999b). At physiological (~0 mV) tonoplast potential Spm and Spd block was approximately 10-fold weaker than that of the FV channel, but the affinity of both channels for Put was fairly comparable (Figure 1, Table 3).

Tonoplast also possesses highly K^+^-selective channels (VK), belonging to so called two-pore K^+^ channels family, TPK (Gobert et al., 2007; Isayenkov et al., 2010). These channels are very abundant in guard cells, where they play an important role in mediation of the vacuolar K^+^ release (Ward and Schroeder, 1994). TPK channels may be also found in other tissues (Pottosin et al., 2003; Gobert et al., 2007). TPK is only weakly sensitive to Spm and Spd (*K_d_* ~1 mM) and practically insensitive to the Put (Hamamoto et al., 2008). Thus, accumulation of PAs during salt stress would primarily inhibit the activity of non-selective cation channels, increasing the overall tonoplast K^+^/Na^+^ selectivity and assisting the efficient vacuolar Na^+^ sequestration (Figure 1).

Effects of PAs on other vacuolar ion transporters are less explored. *Arabidopsis* mutant, lacking the Spm- and tSpm-synthases, consequently has zero Spm and tSpm levels. This mutant shows generally non-altered phenotype under normal growing conditions, except a reduced stem growth (dwarfism) due to the lack of tSpm (Imai et al., 2004). Yet it was hypersensitive to high NaCl and KCl, but not to the equivalent osmotic stress or high MgCl_2_ (Yamaguchi et al., 2006). These mutants also have shown symptoms of the Ca^2+^-deficiency, similar to plants overexpressing vacuolar cation/H^+^ exchangers (CAX). Indeed, transgenic Spm-deficient plants have shown overexpression of several vacuolar CAXs (Figure 1) but same levels of expression of components of the SOS signaling cascade, responsible for the vacuolar Na^+^ sequestration. Causal relations between Spm, CAX-expression, and Ca^2+^ signaling during salt stress remain to be elucidated. Interestingly, whereas Put and Spd but not Spm were essential for the normal growth of *Arabidopsis*, in the case of growth under stress conditions it was just the other way around (Kusano et al., 2008). High ratio of polyamines to diamines positively correlated with a higher activity of vacuolar H^+^-ATPase and PPase as well as with a higher level of phospholipids and lower level of galactolipids in the tonoplast under salt stress (Sun et al., 2002; Liu et al., 2006). In addition to the interaction of PAs with tonoplast phospholipids, binding of PAs to the tonoplast correlated with a higher activity of the V-type H^+^-ATPase and vacuolar Na^+^/H^+^ exchanger, conferring salt tolerance (Zhao and Qin, 2004). Already mentioned tSpm appears to have specific roles in the stem elongation, preventing premature cell death of developing xylem elements (Kakehi et al., 2008; Takahashi and Kakehi, 2010). On the contrary, the maturation of the xylem elements is achieved via PAs exodus to the apoplast and catabolization therein; released H_2_O_2_ caused coordinated stiffening of cell walls as well as programmed cell death (PCD) of xylem elements, due to the induction of a Ca^2+^-permeable conductance in their PM (Tisi et al., 2011; see also the section devoted to PAs and ROS cross-talks below).

At the same time, inhibitory effects of PAs on the vacuolar H^+^-pump activity were also reported (Tang and Newton, 2005; Janicka-Russak et al., 2010). Interestingly, the steady state H^+^ pumping was decreased by PAs, but the V-H^+^-ATPase activity was not significantly affected (Janicka-Russak et al., 2010). This may imply that PAs act as uncouplers. In addition, transport of uncharged PAs across the membrane and their protonation–deprotonation reactions may affect the pH buffering capacity in an acidic compartment and eventually affect the H^+^-ATPase or ATP-synthase activity, as it was shown for the F-type H^+^-ATPase in thylakoids (Ioannidis et al., 2006).

## Modulation of plasma membrane potassium and non-selective channels by polyamines

In animal cells, PAs cause a strongly voltage-dependent block of the inward-rectifying (Kir) K^+^ channels from the intracellular side; in fact, blockage by PAs is the main cause of the channel inward rectification (Lopatin et al., 1994; Kurata et al., 2010). Inward-rectifying K^+^ channels (KIRC) in plants are not related to Kir animal channels and belong to the *Shaker* family, which in animals encode only depolarization-activated K^+^ channels (see Sharma et al., 2013a, for a review). It is not surprising, therefore, that the mechanisms of action of PAs on KIRC may differ from those on Kir. Liu et al. (2000) found that Spm, Spd, and Put, with a little preference, have inhibited KIRC in the guard cell membrane of *Vicia faba* (Table 3). The same work also reported that these PA were also efficient in inhibiting the major component of inward K^+^ current, encoded by KAT1 channel, in a heterologic system. The effect of PAs was voltage-independent and showed the same dose-dependence as inhibition of stomata movements. *In planta* measurements revealed that under drought conditions Spd level increased to levels above 1 mM, whereas Put and Spm levels were lower and practically unchanged. This data was interpreted as the evidence for Spd-induced stomata closure to reduce water loss under stress conditions. Importantly, Spd was only efficient from the interior of the guard cell. Yet, when Spd was added at the cytosolic side of small excised membrane patches, no effect on the single channel activity was observed (Liu et al., 2000). Thus, Spd effect on the KIRC was most likely *indirect* and mediated by some unknown intracellular factor or signaling pathway. On contrary, KIRC in barley roots was only affected by PAs from the *extracellular* side (Zhao et al., 2007). In addition to KIRC, the outward-rectifying K^+^ channel (KORC) was inhibited indiscriminately by Put or Spm (Table 3). These channels are widely present in root cortex and epidermis and encoded (in Arabidopsis) by the GORK gene (Mäser et al., 2001). It should be noted that GORK channel in *Vicia faba* guard cells was unaffected by PAs (Liu et al., 2000). Taken together with a great variability of the PA effects on KORC (e.g., an order of magnitude difference between samples; an occasional but not compulsory reversibility of inhibition) observed in our experiments, it is plausible to suggest that PAs effects on plant *Shaker* K^+^ channels are indirect and can be mediated by different factors, present in the apoplast and/or in the cytosol.

In addition to K^+^ channels, plants express a variety of non-selective cation currents in the PM (see Demidchik and Maathuis, 2007, for a review). The most common voltage-independent non-selective cation current (VI-NSCC) is almost equally permeable for K^+^ and Na^+^, as well as to divalent cations (Ca^2+^). This current is instantaneous and only weakly voltage-dependent (Demidchik and Tester, 2002). In addition to instantaneous currents, mixed non-selective currents with instantaneous and time-dependent outward-rectifying components can be frequently recorded. The time-dependent but not the instantaneous components were sensitive to (inhibited by) external Na^+^. Thus, it was proposed that VI-NSCC in roots and leaves are major mediators of the toxic Na^+^ influx (Shabala et al., 2006). Na^+^-permeable VI-NSCC in roots (Zhao et al., 2007) and leaves (Shabala et al., 2007a,b) were inhibited by externally applied PAs (Table 3). Effect of PAs on the VI-NSCC, albeit reversible, developed slowly (several minutes). There are two possible explanations: (1) PAs acted from the cytosolic side, and their uptake into the cell required a substantial time or (2) PAs effect on the VI-NSCC was indirect. As Zhao et al. (2007) did not found any significant effect of PAs from the cytosolic side on the VI-NSCC, the second possibility seems to be more plausible. Relatively high active concentrations of external PAs raise the question of the physiological significance of their effects on the PM ion channels. However, available data imply that more than half of tissue PAs is associated with the apoplast (Pistocchi et al., 1988 and references therein). Together with high (up to millimolar) levels of PAs, reached at stress conditions, is justifies a relatively high threshold for the PAs effect on the PM channels.

One of the important determinants of the salt sensitivity mechanism is K^+^ loss from plant tissues, caused by the PM depolarization due to the influx of Na^+^ (Shabala and Cuin, 2008). In barley the Na^+^-induced K^+^ efflux is a main cause of the salt sensitivity and a better control of the membrane potential against the depolarization challenge under salinity is crucial for the tolerance (Chen et al., 2007). In pea mesophyll, externally applied PAs not only inhibited the VI-NSCC, but reduced the salt-induced membrane depolarization and associated loss of K^+^ (Shabala et al., 2007a,b). Generalizing this idea, Zepeda-Jazo et al. (2008) proposed a simple model, where PAs inhibition of any NSCC active at depolarized potentials will reduce the membrane depolarization and the loss of K^+^ via GORK and NSCC. GORK inhibition by PAs can further reduce the K^+^ efflux. A prediction of this hypothesis for the NaCl-induced K^+^ efflux was tested on maize and *Arabidopsis* roots. Indeed, PAs could ameliorate NaCl-induced K^+^ efflux in some cases. But, depending on the root zone, growing conditions and PA species, the effect of PAs could be null or even resulted in a strong potentiation of K^+^ efflux (Pandolfi et al., 2010). Obviously, PAs or their catabolites, can cause not only inhibition but also an activation of some cation currents in the PM (see below).

Extracellular application of PAs *per se* induced the membrane depolarization (Di Tomaso et al., 1989; Fromm et al., 1997; Ozawa et al., 2010; Pottosin et al., 2014b), thus, potentially affecting *any* electrogenic transport across the PM and generating driving force for the K^+^ efflux. Our recent pharmacological analysis of the Spm-induced depolarization in barley roots suggested that it was mainly caused by the uptake of PAs via a specific route, not shared with inorganic ions (Pottosin et al., 2014b). Recent advances in the characterization of PA-uptake transporters in plants (Fujita et al., 2012; Mulangi et al., 2012a,b) can provide important clues for the mechanisms of PAs uptake and its impact on the electrogenesis at the plant PM.

## Interplay between polyamines and ROS in the control of passive conductance and pumping atpases of the plasma membrane

**Figure 2:**
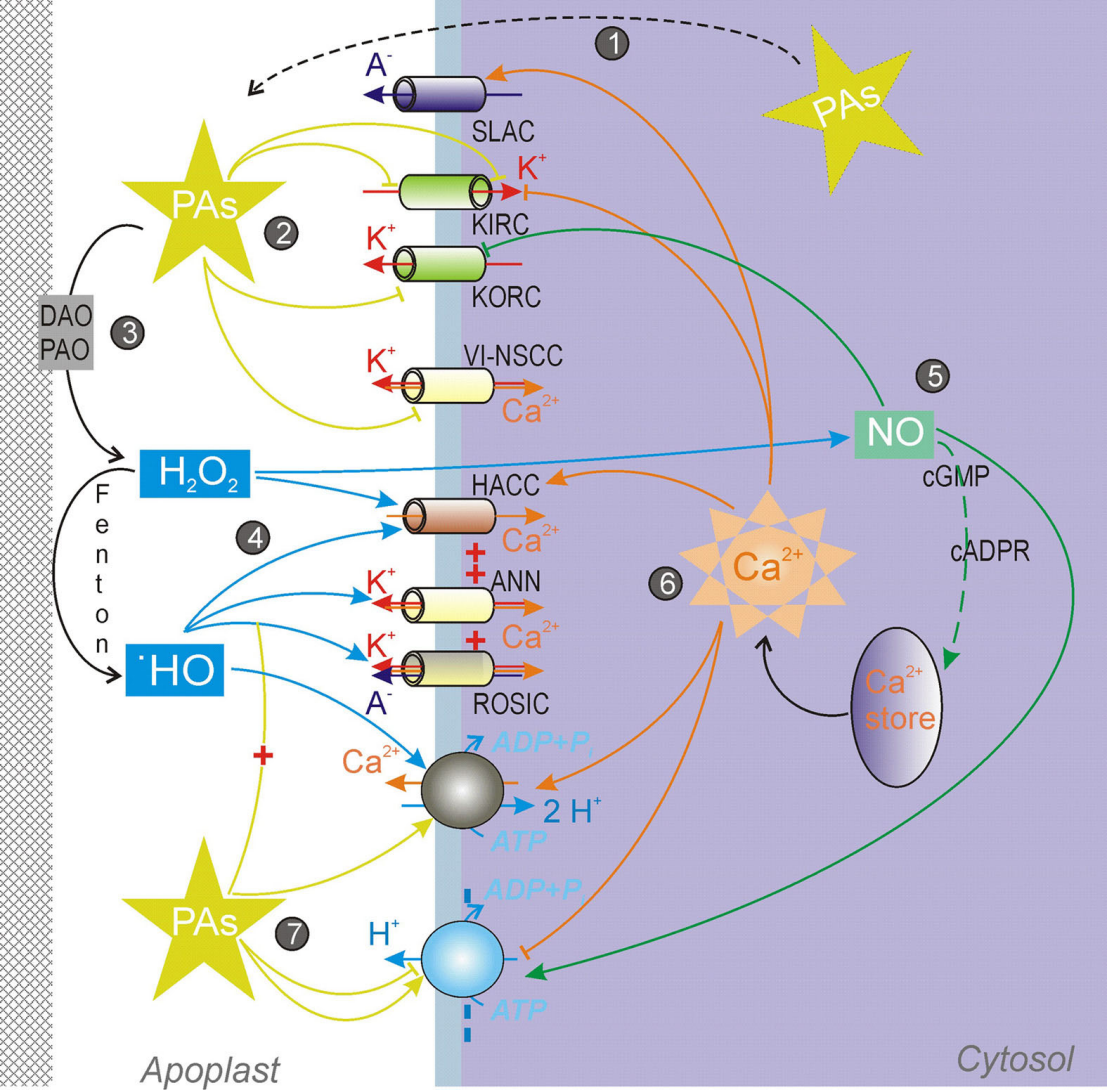
**Regulation of ion transport across the plasma membrane by polyamines and their catabolites. (1)** PAs are exported from the cytosol to the apoplast against the electrochemical gradient. **(2)** PAs inhibit K^+^ (inward-rectifying, KIRC and outward rectifying, KORC) and non-selective voltage-independent cation (VI-NSCC) channels. **(3)** PAs oxidation by diamine (DAO) and/or polyamine (PAO) oxidases generates H_2_O_2_ in the apoplastic space. There H_2_O_2_ can be converted to •OH by the single electron reduction catalyzed by transient valency metal ions. **(4)** H_2_O_2_ and •OH activate a variety of non-selective Ca^2+^-permeable channels, including hyperpolarization-activated Ca^2+^ influx channel (HACC), annexin-formed channel (ANN), and non-selective voltage-independent conductance (ROSIC). **(5)** H_2_O_2,_ released during PAs catabolization, causes a rapid NO generation. In its turn, NO inhibits KORC by a direct nitrosylation and in1duces the intracellular Ca^2+^ release via a pathway involving cGMP and cyclic adenosine ribose (cADPR). **(6)** Ca^2+^-sensitive network. Several PM channels, including slow anion channel (SLAC) and pumps, are regulated by cytosolic Ca^2+^. **(7)** PAs potentiate the ROSIC activation, activate the PM Ca^2+^-ATPase and alter the activity of the PM H^+^-ATPase.

PAs are well-known ROS scavengers and activators of the antioxidant enzymes (Ha et al., 1998; Das and Misra, 2004; Tang and Newton, 2005; Kubiś, 2008; Gill and Tuteja, 2010). At the same time, PAs catabolization generates H_2_O_2_, which can be further converted to different ROS, including the most aggressive one, the hydroxyl radical (•OH). Export of intracellular PAs to the apoplast and their oxidation therein by available diamine (DAO) and/or polyamine (PAO) oxidase to generate H_2_O_2_ (Figure 2) is a common signaling pathway segment, which can be found in a variety of plant adaptive and developmental responses. Depending on the strength of the ROS signal generated by the PAs oxidation, very opposite scenarios—e.g., survival vs. PCD—may be realized (see Moschou and Roubelakis-Angelakis, 2014; Pottosin et al., 2014a, for a recent review).

Studies on transgenic *Arabidopsis* plants, overexpressing enzymes of the Put and Spm biosynthesis, revealed cross-talks between PAs and expression of Ca^2+^-signaling genes, implying a role of PAs in the Ca^2+^ homeostasis and signaling (Marco et al., 2011). A possible link may be via PAs catabolization and ROS-induced Ca^2+^ fluxes. ROS regulate a variety of ion conductances in the PM. Both H_2_O_2_ and •OH activate hyperpolarization-activated Ca^2+^ influx currents (HACC) in roots and leaves (Pei et al., 2000; Demidchik et al., 2003, 2007). It appears that properties of HACC, activated by H_2_O_2_ and •OH, are slightly different, despite sharing some characteristics with constitutive HACC. These channels are usually active at non-physiologically large negative potentials (Demidchik and Maathuis, 2007). The presence of the distinct HACC populations, differently responding to H_2_O_2_ and •OH, is manifested by the fact that in the root mature zone HACC are responding only to •OH, whereas in the distal elongation zone both H_2_O_2_ and •OH could induce the Ca^2+^ influx (Demidchik et al., 2007). •OH can activate a variety of conductances, which not only mediate Ca^2+^ influx, but also cation (K^+^) efflux (Figure 2). One of such conductances is mediated by annexin1. It displays both outward and inward rectifying time-dependent components, mediating K^+^ efflux and Ca^2+^ influx, respectively (Laohavisit et al., 2012). On the other hand, Demidchik et al. (2010) provided the evidence for the •OH-activation of GORK channels, mediating TEA-sensitive K^+^ efflux in *Arabidopsis* roots. However, in pea roots •OH generation caused a rapid cessation of the constitutive GORK (Zepeda-Jazo et al., 2011). Instead, a weakly voltage-dependent instantaneous current, permeable to Ca^2+^, TEA^+^, K^+^, and even small anions (Cl^−^) developed and reached a steady state after 30 min from the initiation of the •OH treatment. Time-dependent depolarization-activated currents developed much later (after 1 h), and were not studied in detail in this plant model. The instantaneous current was termed ROSIC (for ROS-induced conductance) and mediated by tiny (~1 pS conductance) channels (Pottosin et al., 2014a). Both •OH-induced K^+^ efflux in intact pea roots and ROSIC were sensitive to a variety of cation and anion channels blockers (Zepeda-Jazo et al., 2011). ROSIC activation induced membrane depolarization but caused a massive K^+^ efflux so that an efflux of anions likely occurred in parallel (Pottosin et al., 2014a,b). A very similar •OH-induced conductance was reported for barley roots (Velarde-Buendía et al., 2012). PAs unexpectedly stimulated ROSIC and •OH-induced K^+^ efflux in intact roots, although by themselves they were incapable to induce any K^+^ efflux (Zepeda-Jazo et al., 2011; Velarde-Buendía et al., 2012). In pea such potentiation by PAs was restricted to the mature root zone and not observed in the elongation zone (Pottosin et al., 2012). This is consistent with the idea of the presence of distinct ROS-activated channels populations in these zones. Even more strikingly, ROSIC potentiation by PAs was much larger in salt-sensitive barley than in a salt tolerant variety (Velarde-Buendía et al., 2012), again corroborating the idea on the crucial role of the K^+^ retention for the salt tolerance (Chen et al., 2007). Importantly, potentiation of the ROSIC by PAs could be demonstrated in isolated root protoplasts, perfused by an artificial intracellular solution, and containing no amine oxidases. Thus, one has to presume that PAs but not their catabolites acted in this case, and that PAs effects on the ROSIC were likely membrane-delimited.

Externally applied PAs also caused a rapid NO generation, which is likely mediated by PAO and DAO with a generation of H_2_O_2_ upstream to the NO (Tun et al., 2006; Wimalasekera et al., 2011). NO caused the inhibition of GORK due to a direct protein nitrosylation (Sokolovski and Blatt, 2004). NO also caused stomata closure, via a pathway mediated by cGMP and cADPR, and leading to a Ca^2+^ release from an intracellulare store (Figure 2; Neill et al., 2002). This Ca^2+^ release causes the inhibition of KIRC and activation of slow anion channels (SLAC), reducing K^+^ uptake and stimulating the anion efflux, respectively; NO did not have any direct effect on these channels (García-Mata et al., 2003). Prolonged (4 days) exposure to NO caused also up to three-fold activation of the PM H^+^-ATPase (Zandonadi et al., 2010).

Activation of the PM Ca^2+^ permeability by ROS and resulting increases of the intracellular free Ca^2+^ could modulate a variety of ion transport processes across the PM (Figure 2). It also exerts a positive feedback regulation on the ROS production by the PM membrane NADPH-oxidase (Takeda et al., 2008). However, ROS and PAs also affected the Ca^2+^ efflux system, namely the PM Ca^2+^-ATPase. It was shown that both •OH and PAs activated eosine-sensitive Ca^2+^ pumping in intact roots (Bose et al., 2011; Zepeda-Jazo et al., 2011; Velarde-Buendía et al., 2012; Velarde-Buendía, 2013). In pea roots the threshold for •OH activation of the Ca^2+^ pump was at least by an order of magnitude lower as compared for that for the ROSIC (Zepeda-Jazo et al., 2011). Ca^2+^ efflux induced by •OH in pea roots was transient but could be potentiated by PAs Spm> Spd >Put (Zepeda-Jazo et al., 2011; Pottosin et al., 2012). In the case of Spm, a long-lasting steady state Ca^2+^ efflux was observed, arguing for non-additive effects of •OH and PAs. No such behavior was found in barley roots, where •OH- and PA-effects on Ca^2+^ efflux were roughly additive (Velarde-Buendía et al., 2012). Velarde-Buendía (2013) demonstrated that in pea roots PAs affected H^+^ fluxes in a differential manner. Whereas Spm caused a net H^+^ influx, Put caused a prolonged vanadate-sensitive H^+^ efflux, caused by the H^+^-ATPase activation. Spm-induced H^+^ influx was consistent with the 1 Ca^2+^: 2 H^+^ exchange mechanism, demonstrated previously (Beffagna et al., 2000). The activation of the H^+^-ATPase by Put appeared to be indirect and coupled to the Ca^2+^-pump activation (Velarde-Buendía, 2013; Pottosin et al., 2014b). As the PM H^+^-ATPase is inhibited by cytosolic Ca^2+^ (Kinoshita et al., 1995; Brault et al., 2004), it is logical to propose that a cross-talk between the two pumps is mediated by the intracellular Ca^2+^ changes. Yet a supposed decrease of the intracellular Ca^2+^ by PAs needs to be demonstrated directly in future experiments.

H^+^-ATPase operates as a powerhouse, controlling the electric potential difference and the active ion exchange across the PM (Palmgren, 2001). Existing data on immediate effects of PAs on the H^+^-ATPase pumping activity are controversial, with both activation (in rice, Reggiani et al., 1992 and wheat, Liu et al., 2005) and inhibition (in maize, Pandolfi et al., 2010) effects reported. Garufi et al. (2007) described a very specific mechanism of the H^+^-ATPase activation by intracellular Spm, but not by Spd or Put. Spm promoted the binding of 14-3-3 proteins to the unphosphorylated H^+^-ATPase, thus increasing its hydrolytic activity. Long-term treatment with PAs appears to increase the activity of the PM Ca^2+^-ATPase (Sudha and Ravishankar, 2003) and reduced the magnitude of changes in the H^+^-ATPase activity, induced by the salt stress (Sun et al., 2002; Roy et al., 2005; Tang and Newton, 2005). One day incubation with PAs caused a decrease in transcripts of one of the H^+^-ATPase isoforms, resulting in substantially decreased H^+^ pumping across the PM (Janicka-Russak et al., 2010).

## Implications for stress responses: current stand and perspective

Global climate change is expected to increase the frequency and severity of drought and flooding events in many regions world-wide (Setter and Waters, 2003; Voesenek and Sasidharan, 2013), severely affecting the crop production. On a global scale, the overall loss in food and fiber production due to abiotic stresses is estimated at US$120 billion p.a. and predicted to increase (http://www.fao.org/docrep/008/y5800e/Y5800E06.htm). Also, global availability of good quality water is also becoming a limiting and increasingly expensive resource, and the cost of irrigation-induced salinity is estimated to exceed US$11 billion p.a. (Shabala, 2013). Thus, understanding the role of PAs in plant adaptive responses to drought, salinity and flooding may be instrumental in breeding crops with improved tolerance to these stresses to overcome the above losses.

### Drought stress

Multiple mechanisms contribute to plant adaptive responses to drought. The major ones include (Hu and Schmidhalter, 2005; Shabala and Pottosin, 2014): better stomata control and reduced transpiration under drought conditions; efficient osmotic adjustment, and maintenance of the turgor pressure; prevention of the drought-induced accumulation of ROS; improved water use efficiency and control of a long-distance water transport in plants; maintaining optimal energy status; and improved leaf photochemistry via maintenance of the intracellular ionic homeostasis and charge balance. For obvious reasons, each of these mechanisms is intrinsically dependent on membrane transport activity and implies efficient regulation of ion channels and transporters under stress conditions. Indeed, stomatal opening and closure are strongly dependent on the rapid movement of K^+^ into and out of the guard cell (Blatt, 2000). As commented above, the ability of PAs to inhibit KIRC in the guard cell membrane of *Vicia faba* (Liu et al., 2000) may be essential to prevent stomatal opening under drought conditions, thus reducing transpirational water losses. Consistent with this notion, Arabidopsis *gork-1* mutant plants lacking functional GORK channels showed much poorer performance under drought stress, due to their inability to close stomata (Hosy et al., 2003). These findings are in a good agreement with the fact that PAs inhibit opening and induce closure of stomata (Liu et al., 2000; Shi et al., 2010). Importantly, KIRC inhibition by the Spd in *Vicia* guard cells occurred only from the cytosolic side (Liu et al., 2000) highlighting the importance of PA compartmentation.

Drought stress also results in a rapid increase in the level of ROS species in plant tissues (Miller et al., 2010). Polyamines may play a dual role in the process. First, PA may play a critical role in drought stress signaling to confer adaptive responses. According to suggested models, drought-induced increase in ABA content may promote PAs accumulation and exodus into the apoplast, where they were oxidized by the apoplastic amine oxidases, producing H_2_O_2_ to be used in the signaling cascade (Toumi et al., 2010). On the other hand, PAs are known to significantly enhance activity of both enzymatic (Shi et al., 2010; Wang et al., 2011; Radhakrishnan and Lee, 2013) and non-enzymatic (Högy et al., 2010; Asthir et al., 2012) antioxidants. Therefore, the PA control over the balance between ROS production and scavenging may “shape” H_2_O_2_ signal, conferring differential stress responses between species and genotypes. Again, tissue- and organelle-specificity of PA accumulation is absolutely essential for this process. Also, given the fact that different PAs may have a different potency for H_2_O_2_ production and ROS scavenging, it is not the absolute quantity but a balance between various PAs that may be critical to determine the cell fate. Consistent with this notion, An et al. (2012) recently showed that the extent of membrane damage by drought in maize was dependent on Spd+Spm/Put ratio in plant tissues.

Another important signaling component potentially related to changes in PAs levels is the stress-induced Ca^2+^ signature. Stress- or stimuli-induced elevations in cytosolic free Ca^2+^, [Ca^2+^]_cyt_, vary in their magnitude, frequency, and shape. These depend on the severity of the stress as well the type of stress experienced, thus creating a unique stress-specific calcium “signature” that is then decoded by signal transduction networks (Bose et al., 2011). The drought stress is not an exception, and transient elevations in cytosolic free Ca^2+^ were reported in response to both hyperosmotic (Ng et al., 2001) and ABA (McAinsh et al., 1997) treatments. As prolonged [Ca^2+^]_cyt_ elevation is detrimental to normal cell metabolism, the basal [Ca^2+^]_cyt_ levels must be restored after the signaling process has been completed. This implies involvement of active Ca^2+^ efflux systems, such as PM and endomembrane Ca^2+^-ATPase pumps and exchangers (Bose et al., 2011). Meanwhile, Ca^2+^ efflux was shown to be induced by •OH and potentiated by PAs in a sequence Spm > Spd > Put (Zepeda-Jazo et al., 2011; Pottosin et al., 2012). Thus, the interplay between tissue-specific ROS and PA production and accumulation may confer the shape of stress-specific Ca^2+^ signatures via the modulation of the Ca^2+^-ATPase activity.

### Salinity stress

Plant salinity stress tolerance is a polygenic trait conferred by a large number of sub-traits; each of these is ultimately related to the regulation of membrane-transport activity and ionic homeostasis. The major traits include (Shabala and Munns, 2012): osmotic adjustment; Na^+^ exclusion from uptake by roots; intracellular Na^+^ sequestration; K^+^ retention in the cytosol; tissue-specific Na^+^ sequestration; control of the xylem ion loading; Na^+^ removal from the shoot; and oxidative stress tolerance. Many if not all these traits may be causally related to, and controlled by, salinity-induced changes in PA levels in various cells compartments.

Rapid osmotic adjustment is absolutely critical to maintain cell turgor and support expansion growth of roots and shoots under saline conditions. Accumulation of K^+^ plays a pivotal role in this process, contributing on average to between 35 and 50% of the cell osmotic potential in crops (Shabala and Pottosin, 2014). At the same time, high intracellular K^+^ concentrations are required to determine the cell fate and its transition to the PCD. The loss of potassium has been shown to play a primary role in cell shrinkage, caspase activation, and nuclease activity during apoptosis (one of the forms of PCD) in both mammalian (Hughes and Cidlowski, 1999) and plant (Shabala et al., 2007a,b) systems. In this context, the observation that outward-rectifying K^+^ channels in root epidermis were inhibited by Put or Spm (Table 3) may be considered as an essential trait enabling K^+^ retention in the root and thus contributing to both osmotic adjustment and cell fate determination under saline conditions.

Both changes in the expression levels and activation of existing proteins involved in K^+^ transport and sequestration are essential for maintenance in cytosolic K^+^ homeostasis under saline conditions. For example, AtCHX17, a member of the CPA2 family of transporters was found to be strongly induced by salinity (Kreps et al., 2002; Cellier et al., 2004), to compensate for NaCl-induced K^+^ exodus from the cytosol resulting from salt-induced depolarization of the PM. However, as the transport capacity of high-affinity K^+^ transporters is about 3 orders of magnitude lower compared with channels (Shabala and Pottosin, 2014), cytosolic K^+^ homeostasis is seriously compromised. Thus, post-translational regulation and modulation of activities of existing channels or transporters by various factors and second messengers (including PAs) is more significant. More details on factors controlling K^+^ transport under stress conditions are available in Shabala and Pottosin (2014).

Efficient vacuolar sequestration of the cytotoxic Na^+^ is another prominent mechanism conferring salinity tolerance in plants. This sequestration is achieved by mean of the tonoplast Na^+^/H^+^ antiporters fueled by the vacuolar H^+^-ATPase and H^+^-PPase pumps (Hasegawa, 2013; Shabala, 2013). In addition, toxic Na^+^ ions must be prevented from leaking back into cytosol. Thus, to avoid energy consuming futile Na^+^ cycling between cytosol and vacuole and to achieve efficient vacuolar sequestration of toxic Na^+^, passive tonoplast Na^+^ conductance has to be kept at absolute minimum. This implies a strict and efficient control over Na^+^-permeable tonoplast SV and FV channels (Bonales-Alatorre et al., 2013). Each of these channels is PA-sensitive and can be blocked by the physiologically relevant concentrations of PAs (see Figure 1). Therefore, salinity stress-induced elevation in PA levels may be essential to enable efficient vacuolar Na^+^ sequestration. Consistent with this notion, salt stress inhibited the activity of polyamine synthesizing enzymes (L-arginine decarboxylase and L-ornithine decarboxylase) in glycophyte species of *Vigna radiata* but not in halophyte *P. undulate* (Friedman et al., 1989), thus potentially enabling efficient control over vacuolar Na^+^ sequestration in the latter (naturally salt tolerant) species.

Reducing net Na^+^ accumulation in the cytosol by controlling the rate of its transport across the PM may be another way of improving plant performance under saline conditions. Non-selective cation channels (NSCC) are considered to be a major pathway of Na^+^ uptake into the cell (Demidchik and Maathuis, 2007), and physiologically relevant concentrations of PAs were efficient in inhibiting NSCC-mediated Na^+^ currents in leaf (Shabala et al., 2007a,b) and root (Zhao et al., 2007; Zepeda-Jazo et al., 2008) tissues.

### Flooding stress

Two major factors affect plant growth and performance in flooded soils: reduced oxygen availability leading to a sharp decline in ATP production, and elemental toxicity originating from the changes in the soil redox potential (Shabala, 2011). Acclimation to flooded conditions requires significant metabolic alterations in living cells. This includes reduced energy consumption, activation of pathways that generate ATP without oxidative phosphorylation, and increased chaperone activity to deal with increased ROS production (Bailey-Serres and Voesenek, 2010; Voesenek and Sasidharan, 2013). Polyamines seem to be instrumental in this metabolic adaptation. Accumulation of Put in flooded roots was shown to be able to stimulate the PM ATPase activity (Bertani et al., 1997), conferring beneficial effects to cell ionic homeostasis and nutrient acquisition. Superoxide radical and H_2_O_2_ contents were also reduced in flooding-stressed onion plants after Put pre-treatment (Yiu et al., 2009). Jia et al. (2010) showed that application of exogenous Spd to hypoxic cucumber roots or conversion of Put to Spd and Spm enhanced the aerobic respiration but inhibited the fermentation metabolism in roots, leading to an increase in ATP content and alleviation of the stress symptoms.

A massive increase in the amount of available Mn and Fe in the soil solution is observed within a few days of onset of waterlogging, often to above toxic levels (Marschner, 1995; Zeng et al., 2013), due to the changes in the soil redox potential. Being a transition metal, Fe is highly redox active and, in the presence of H_2_O_2_, can mediate production of the hydroxyl radical through the Fenton reaction (Rodrigo-Moreno et al., 2013). This may cause lipid peroxidation and damage to key cellular structures as well as result in a massive K^+^ exodus from the cytosol mediated by •OH-activated K^+^-selective outward rectifying (Demidchik et al., 2010) and non-selective K^+^ permeable (Zepeda-Jazo et al., 2011) channels. Both these processes are detrimental to cell metabolism and plant performance under stress conditions. Importantly, a regulatory role of PAs in plant adaptation to flooding seems to be closely related to intracellular K^+^ homeostasis. In the absence of K^+^, anoxia led to a decrease in Put, Spd, and Spm levels. The presence of K^+^ ions during the anaerobic treatment abolished the negative effect of anoxia on polyamine titers and slightly increased them (Reggiani et al., 1993).

## Outlook

A lesson learned from studies of the PAs effects on plant ion channels is that, contrary to their animal counterparts, a direct pore blockage mechanism is uncommon. A notable exception to that observation is a high affinity block of vacuolar non-selective cation channels of FV and SV types. Because these channels are ubiquitously expressed in plant tissues the model shown in Figure 1 describing the impact of PAs on vacuolar cation transport, can be considered as a general one. Validation of this working model can be demonstrated by genetic manipulation of SV, FV and VK channels by silencing, or site directed mutations, affecting channels' sensitivity to PAs. While the molecular identities of SV and VK channels are known, that of the FV channel remains cryptic. Completing of this lacking information will allow the manipulation of the overall tonoplast cation transport and eventually will help to understand the role of PAs in its control in plant responses to abiotic stresses.

In most cases, the action spectrum of PAs depends on the side of the membrane/ compartment, whether it is vacuolar lumen, cytosol or apoplast. Unfortunately, PAs compartmentation and membrane transport in plants are poorly explored. In particular, mechanisms of PAs uptake or active efflux across the PM are unknown (Igarashi and Kashiwagi, 2010). This knowledge is very important, however, to understand the exodus of PAs to the apoplast, where they are normally absent. In many cases, it can be proved that rather than PAs themselves, their catabolites (and especially ROS), exert the effect on plant membrane transporters. It is important to mention that the apparent specificity of diamine (Put) vs. PAs (Spd, Spm) effects in this case may be caused by a higher activity of the apoplastic DAO in dicots like Fabaceae or PAO in monocots like Poaceae (Moschou et al., 2008). Overall, the relation between PAs biosynthesis and catabolism (or, in other words, respective levels of PAs and their catabolites) may determine whether survival or PCD responses would be initiated (Moschou and Roubelakis-Angelakis, 2014). This is also applicable to the balance between PAs actions as ROS scavengers and antioxidant system activators, and PAs as a ROS source. In this regard, ROS speciation also becomes crucial. Whereas H_2_O_2_ is a relatively long-living and easy membrane-permeable molecule, •OH is short-lived and acts in the closest vicinity of the transient valency metal, which catalyzed its generation. A substantial evidence was obtained for differential effects of •OH and H_2_O_2_ on plant membrane transporters. In particular, a newly described dual cation and anion conductance, ROSIC, is activated only by •OH and PAs further modulate it in species- and tissue-dependent mode.

Apart of recently revealed cross-talks between PAs and ROS, an important link between PAs catabolism and stress response may be the PAs-induced generation of the NO (Wimalasekera et al., 2011), which in turn affects a variety of PM transporters, either directly or via the intracellular Ca^2+^ signal (Figure 2). In addition to ROS-activated Ca^2+^ influx channels, both ROS and PAs are capable to activate PM Ca^2+^ pumps. Thus, the fine tuning of Ca^2+^ signal may be achieved, which is worth of further experimental exploration.

It is conceivable that stress-induced changes of PAs and ROS metabolism were adapted for the stress resistance in a rather opportunistic way. Whereas few direct sensors for PAs and ROS evolved, other targets may be indirect and the net effect, e.g., on the K^+^ transport across the PM, may be rather variable (Pandolfi et al., 2010). While searching for a solution of the equation with many parameters, one needs to take into the account PAs synthesis, transport, and catabolization. In addition, tissue-, species-, and physiological status-dependent expression of different ion channels and transporters as well as the modes of action of PAs and their catabolites should be also always kept in mind. Despite its complexity, this task is the only possible alternative, as the “spray and pray” strategy seems to be not applicable in the case of polyamines.

### Conflict of interest statement

The authors declare that the research was conducted in the absence of any commercial or financial relationships that could be construed as a potential conflict of interest.
